# Lipidomic analyses of five *Carya illinoinensis* cultivars

**DOI:** 10.1002/fsn3.3572

**Published:** 2023-07-31

**Authors:** Zhe Zhao, Fei Wang, Tian Hu, Chun‐hua Zhou

**Affiliations:** ^1^ College of Horticulture and Landscape Architecture Yangzhou University Yangzhou China

**Keywords:** *Carya illinoinensis*, cultivars, kernel, lipidomics, nutrients

## Abstract

*Carya illinoinensis* (Wangenh.) K. Koch, nuts are a renowned health food. However, there are many cultivars of this nut tree, and their mature kernel lipid composition has not been thoroughly studied. Therefore, we used liquid chromatography–mass spectrometry (LC–MS) to analyze the lipid composition of mature nuts of five *C. illinoinensis* cultivars*.* In the mature kernels of all cultivars, there were 58 lipid types which were mainly composed of glycerolipids (c. 65%) and phospholipids (>30%). Triacylglycerol (TG) accounted for the largest proportion of mature nuts of all cultivars, exceeding 50%; and diacylglycerol (DG), ceramide (Cer), phosphatidylcholine (PC), and phosphatidylethanolamine (PE) were also relatively high. Additionally, nuts contain fatty acids, mainly oleic, linoleic, and linolenic acids. Our research provides a new perspective for the processing and utilization of plant and edible oils, and for the use of *C. illinoinensis* kernels in the development of medicine and food science.

## Graphical abstract

*Carya illinoinensis* (Wangenh.) K. Koch, nuts are a renowned health food. However, there are many cultivars of this nut tree, and their mature kernel lipid composition has not been thoroughly studied. Therefore, we used liquid chromatography–mass spectrometry (LC–MS) to analyze the lipid composition of mature nuts of five *C. illinoinensis* cultivars. In the mature kernels of all cultivars, there were 58 lipid types which were mainly composed of glycerolipids (c. 65%) and phospholipids (>30%). TG accounted for the largest proportion of mature nuts of all cultivars, exceeding 50%; and DG, Cer, PC, and PE were also relatively high. Additionally, nuts contain fatty acids, mainly oleic, linoleic, and linolenic acids. Our research provides a new perspective for the processing and utilization of plant and edible oils, and for the use of *C. illinoinensis* kernels in the development of medicine and food science.

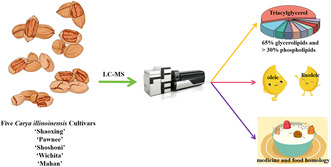

## 1 INTRODUCTION

*Carya illinoinensis* is a widely recognized woody oil crop, with high nutritional value and economic benefits (Yuhan et al., 2022). Compared with other oil crops such as olive and oil peony, the *C. illinoinensis* kernel is edible and easy to obtain. It can be used for making dessert, candy, or ice cream, and the oil extracted can be used for medicine and essential oil (Du et al., 2022). Moreover, the kernel is rich in nutrients, and 70%–75% of it is oil (Zhang et al., 2022). Notably, *C. illinoinensis* nuts are often eaten as food, as they have been proven to have beneficial effects on cardiovascular diseases (Concilco‐Alberto et al., 2022). But the lipid composition of the *C. illinoinensis* ripe kernel remains unknown and so revealing its composition might realize beneficial medical and food science outcomes.

As the most important component of vegetable oil, lipids can be divided into eight categories according to their structure and biochemical properties, namely, fatty acid (FA), glycerolipids (GLs), glycerophospholipids (GPs), sphingolipids (SPs), saccharolipids (SLs), sterol lipids (STs), polyketides (PKs), and prenol lipids (PRs; Yu et al., 2020). Lipids play an important role in organism growth and development, energy storage, material transport, and the recognition and transduction of molecular signals (Belayneh et al., 2018). In the human body, lipids as important nutrients not only provide required nutrition and energy but also participate in immune regulation and inflammatory response, playing an important role in maintaining health (Velásquez et al., 2015). Lipid metabolism is closely related to disease/infection occurrence and development, and preliminary diagnosis and assessment can be made by analyzing lipid changes.

Lipidomics is widely used, and especially prominent in medical and food treatment. Determining changes in lipid type and content in various body organs enables disease conditions to be evaluated at an early stage of occurrence (Gold et al., 2022). Analysis of lipid composition and relative content changes can provide information for clarifying tumor formation mechanisms, and play a role in preventing cardiovascular and cerebrovascular diseases through drug research and development (Ramos & Meeusen, 2022). In food science, lipidomics can be used for quality identification, through the analysis of lipid types and content, to find characteristic differences and screen out altered products. It can also be used to trace lipid origin by analyzing lipid metabolism and lipid molecular regulation mechanisms in food samples, as products with the same mechanism can be used as raw materials to improve existing products (Zhou et al., 2022).

Lipid composition is often different between foods or oil crops, which will affect the function and characteristics of the substance itself. The main component of edible oil is lipid, and the lipid composition and oil content squeezed from different oil crops are obviously different. In recent years, there have been many studies on lipid profiling in woody oil crops using lipidomics. Li et al. (2022) analyzed camellia seed oil lipidomics, and detected 278 lipid components in total, and compared with other woody oils such as hazelnut and olive oil, it was also rich in glycerolipids, with 221 kinds. Furthermore, the functional characteristics of each part were revealed by lipid classification, showing that camellia seed oil had significant prevention effects on cardiovascular and cerebrovascular diseases and inhibited fat accumulation in vivo. Huang et al. (2021) analyzed lipid composition and content during *Carya cathayensis* embryonic development and found that mature kernels contained 544 kinds of lipids. Moreover, the proportion of glycerophospholipids in mature hickory kernels was relatively the largest, accounting for 59.94%. Song et al. (2018) analyzed the phospholipid content of six nuts using lipidomics and found that although peanut was rich in phospholipid species, the actual phospholipid content was very low, while almond and pecan had low total phospholipid species and concentration, but high total unsaturated fatty acid content. He concluded that individual lipids do not determine the overall nutritional value of whole nuts. However, in the analysis of the lipid composition of nuts and woody oil crops, there are relatively few reports on the lipid metabolism of *C. illinoinensis*. Therefore, it is urgent to address this shortcoming to supplement our knowledge of the lipid composition of economic forest crops.

After the oil was extracted from mature *C. illinoinensis* kernels, we conducted lipidomics analysis to provide a lipid profile and reveal its unique properties and functions through a qualitative analysis of lipid type and content (Chung et al., 2017). Furthermore, lipidomics analysis of *C. illinoinensis* is also helpful for the popularization and utilization of its oil and the development of high‐end oil products.

Our main objective was to elucidate the lipid composition of mature nuts of five *C. illinoinensis* cultivars using liquid chromatography–mass spectrometry (LC–MS). The method included: (1) quantitative analysis of lipid structure characteristics and different lipid contents; (2) evaluation of the degree of correlation of lipids; and (3) analysis of different lipids and their functional enrichment among the five cultivars. Through this research, we can deepen our understanding of the lipid composition of mature *C. illinoinensis* kernels and provide a theoretical basis for kernel development in health care and edible oil processing and utilization (Xie et al., 2023).

## 2 MATERIALS AND METHODS

### 2.1 Plant material

Plant samples were collected from a commercial orchard in Heyue Park, Baoying County, Yangzhou City, Jiangsu Province, China. In the park, the average annual air temperature is 14.4°C, the average annual precipitation is 1000 mm, and the relative humidity is 71%. In the commercial orchards, three trees of each cultivar with good and uniform growth conditions were randomly selected as sample trees. Mature kernel samples of five cultivars (‘Shaoxing’, ‘Pawnee’, ‘Shoshoni’, ‘Wichita’, and ‘Mahan’) were collected from the east, south, west, and north of each tree. One sample from each of the four cardinal directions was collected for every tree, and 12 fruits were collected. For each cultivar, three samples were randomly selected from the 12 fruits collected as three replicates in subsequent analyses. The fresh fruits (samples) were put into an ice box and taken back to the laboratory to be stored in a refrigerator at −80°C. Fruits of each cultivar were treated and analyzed every other day.

### 2.2 Chemicals

LC–MS grade isopropyl alcohol (IPA) and methanol (MeOH) were purchased from Fisher Scientific. Chloroform was obtained from Sinopharm. Ultrapure water was generated using a Milli‐Q system. Acetonitrile (ACN) was purchased from Fisher Scientific, formic acid from TCI, and ammonium formate from Sigma‐Aldrich.

### 2.3 Instruments

A high‐speed centrifuge was obtained from Hunan Xiangyi Experiment Equipment Co., Ltd., a Vortex mixer from Haimen Kylin‐bell Lab Instruments Co., Ltd., and a Centrifugal vacuum evaporator from Eppendorf China Ltd.. A tissue grinder was obtained from Zhejiang Meibi Experiment Equipment Co., Ltd., and microporous membrane filters (0.22 μm) from Tianjin Jinteng Experiment Equipment Co., Ltd. The LC analysis was performed on a Vanquish UHPLC System (Thermo Fisher Scientific), and mass spectrometric detection of metabolites was performed on a Q Exactive (Thermo Fisher Scientific).

### 2.4 Total lipid extraction

An appropriate sample amount was placed in a 2‐mL EP tube, 750 μL of mixed solvent (chloroform: methanol, 2:1, v/v) added at −20°C, and vortexed for 30 s. Two steel balls were placed in the tissue grinder, and ground for 60 s at 50 Hz, and repeated twice. After leaving the tube on ice for 40 min, we added 190 μL H_2_O, vortexed it for 30 s, and incubated it on ice for 10 min. It was then centrifuged at 10,200 *g* for 5 min at room temperature and a 300‐μL organic layer was transferred into a new centrifuge tube. We then added 500 μL of mixed solvent (chloroform: methanol, 2:1, v/v), vortexed it for 30 s, and centrifuged it at 10,200 *g* for 5 min at room temperature and transferred a 400‐μL organic layer into the same centrifuge tube. Samples were concentrated to dryness in a vacuum (Huang et al., 2021), then dissolved in 200‐μL isopropanol, and the supernatant was filtered through a 0.22‐μm membrane to obtain the prepared samples for LC–MS.

### 2.5 LC–MS analysis

Chromatographic condition (Dalli et al., 2018): Chromatographic separation was used with an ACQUITY UPLC® BEH C18 (2.1 × 100 mm, 1.7 μm, Waters) column maintained at 50°C. The temperature of the autosampler was 8°C. Gradient elution of analytes was carried out with acetonitrile: water = 60: 40 (0.1% formic acid + 10 mM ammonium formate) (A2) and isopropanol: acetonitrile = 90: 10 (0.1% formic acid + 10 mM ammonium formate) (B2) at a flow rate of 0.25 mL/min, 2 μL was injected for gradient elution. Separation was conducted under the following gradient: 0–5 min, 57%–70% A2; 5–5.1 min, 50%–57% A2; 5.1–14 min, 30%–50% A2; 14–14.1 min, 30% A2; 14.1–21 min, 1%–30% A2; 21–24 min, 1% A2; 24–24.1 min, 1%–70% A2; 24.1–28 min, 70% A2.

Mass spectrum condition (Dasilva et al., 2019): The ESI‐MSn experiments were conducted with a spray voltage of 3.5 and 2.5 kV in positive and negative modes, respectively. Sheath gas and auxiliary gas were set at 30 and 10 arbitrary units, respectively, and the capillary temperature was 325°C. The orbitrap analyzer scanned over a mass range of m/z 150–2000 for full scan at a mass resolution of 35,000. Data‐dependent acquisition (DDA) MS/MS experiments were performed with HCD scan. The normalized collision energy was 30 eV, and dynamic exclusion was implemented to remove some unnecessary information in MS/MS spectra.

## 3 RESULTS AND DISCUSSION

### 3.1 Quantitative analysis of lipids

Kernel class and composition of the five *Carya illinoinensis* cultivars were compared and analyzed. There were 58 kinds of lipids, and triglyceride (TG) had the highest content in each variety (Table 1). In this lipid, there were 875, 868, 866, 877, and 877 substances detected in ‘Mahan’, ‘Pawnee’, ‘Shoshoni’, ‘Wichita’, and ‘Shaoxing’ cultivars, respectively. Triglyceride, as an important index in human blood lipid examination, plays a significant role in heat supply, liver protection, human blood dynamic balance maintenance, and atherosclerosis prevention. DG had the second highest lipid content in mature nuts, containing 194, 190, 193, 193, and 190 substances, respectively. DG can stimulate fat utilization in humans, decompose more energy, effectively promote human metabolism after meals, and reduce visceral fat accumulation. Both DG and TG are glycerolipids, which are often considered as the main component of edible oil. The high content and large proportion of glycerolipids in *C. illinoinensis* kernels indicate strong potential in oil extraction and edible oil processing and utilization. In view of its bodily function role, *C. illinoinensis* oil could help prevent obesity and reduce weight and fat. At the same time, glycerolipids often cause environmental pollution from the chemical preparation and extraction process. Therefore, using biological methods to extract glycerolipids from fruit trees with high glycerolipid content such as *C. illinoinensis* could realize green and environment‐friendly solutions.

**TABLE 1:** Composition of lipids in mature nuts of five *Carya illinoinensis* cultivars.

Lipid classification	Number of substances in each lipid variety				
	Pawnee	Mahan	Shaoxing	Wichita	Shoshoni
ZyE	15	15	15	15	15
WE	8	9	9	8	8
TG	868	875	877	877	866
StE	9	9	9	9	9
ST	1	1	1	1	1
SQDG	34	33	34	34	34
SPHP	1	1	0	0	0
SPH	2	4	4	3	4
SM	4	4	4	4	4
SiE	4	4	4	4	3
PS	61	61	62	62	61
PMe	30	30	30	30	30
PIP3	2	2	2	2	2
PIP2	9	9	9	9	9
PIP	9	10	10	10	10
PI	66	66	66	66	66
PG	55	54	53	54	53
PEt	15	15	15	15	15
PE	68	67	68	68	68
PC	74	74	73	74	74
PA	53	53	53	53	53
MLCL	13	13	13	13	13
MGMG	3	3	3	3	3
MGDG	31	33	32	33	33
MG	8	8	8	8	8
MePC	13	13	13	12	13
LPMe	3	2	2	3	3
LPI	5	5	5	5	5
LPG	3	3	3	3	3
LPE	7	7	7	7	7
LPC	5	5	3	5	5
LdMePE	2	2	2	2	2
LBPA	6	6	6	6	6
Hex3Cer	2	2	3	2	2
Hex2Cer	4	4	4	4	4
Hex1Cer	40	39	39	40	39
GM3	2	2	2	2	2
dMePE	15	15	14	15	14
DLCL	3	3	3	3	3
DGMG	5	3	3	4	3
DGDG	53	52	50	53	50
DG	190	194	190	193	193
cPA	4	4	4	4	4
Co	3	3	3	3	3
CmE	2	2	2	2	2
CL	45	44	44	44	42
ChE	1	1	1	1	1
CerP	1	2	2	2	2
CerG3GNAc1	3	3	3	3	3
CerG2GNAc1	2	2	2	2	2
Cer	75	77	77	77	78
BisMePA	67	66	67	66	66
BiotinylPE	2	2	2	2	2
AcHexZyE	11	11	11	11	11
AcHexStE	7	7	7	7	7
AcHexSiE	16	16	16	16	16
AcHexCmE	6	6	6	6	6
AcHexChE	12	12	12	12	12

Sphingolipids are often used as molecules in regulating plant signal transduction. In the mature kernels of five *C. illinoinensis* cultivars, the number of sphingolipid subclasses and substances was relatively low. Only two to four SM and SPH substances were detected in each variety, only one SPHP substance was detected in ‘Mahan’ and ‘Pawnee’, respectively, and no such lipids were detected in the other three cultivars.

**FIGURE 1:**
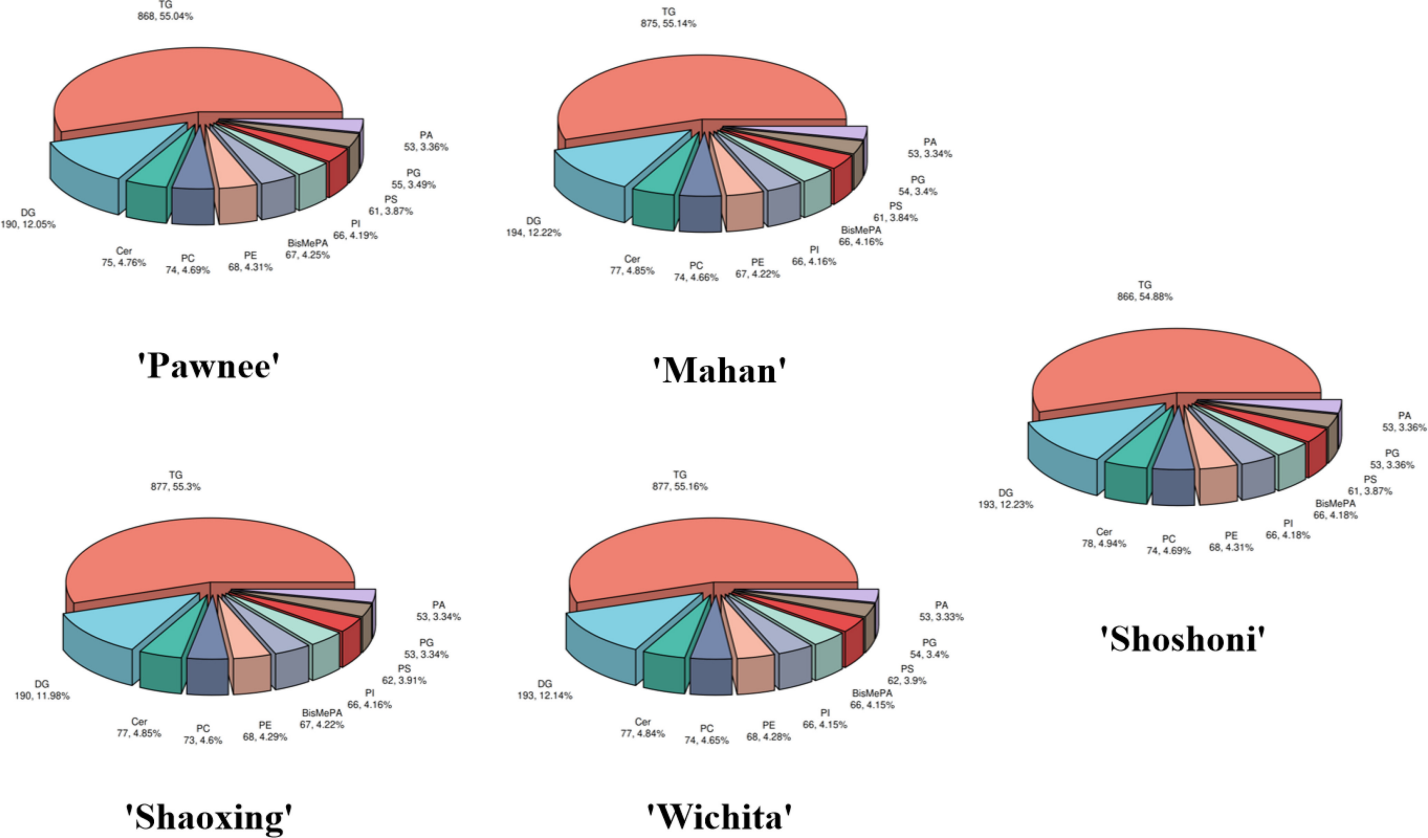
Pie chart of lipid Class classification in five cultivars. The lipids of each cultivar were classified according to their lipid structure, each color area in the pie chart represents the structure of different types of lipids, and numbers in the figure indicate the number of substances of the lipid, the percentage is the proportion.

There were no significant differences in lipid composition among cultivars (Figure 1). TG accounted for the largest proportion of mature nuts, exceeding 50% in all cultivars. DG, Cer, PC, and PE ranked second to fifth in lipid content, respectively.

Quantitative analysis of lipids in mature nuts of five *Carya illinoinensis* cultivars showed that lipids in ‘Mahan’, ‘Pawnee’, ‘Shoshoni’, ‘Wichita’, and ‘Shaoxing’ were mainly composed of glycerolipids and phospholipids, where glycerolipids accounted for c. 65% and phospholipids accounted for >30%. In plants, phospholipids and glycerolipids mainly exist in oilseeds, where they are an important component, indicating that *C. illinoinensis* kernels have potential in oil extraction and oil production. Additionally, glycerolipids and phospholipids have significant effects promoting fat metabolism, reducing serum cholesterol, and improving blood circulation, reflecting a key role in health care and perhaps further development in future medicine and food homology studies.

### 3.2 Hierarchical cluster tree of population sample

**FIGURE 2:**
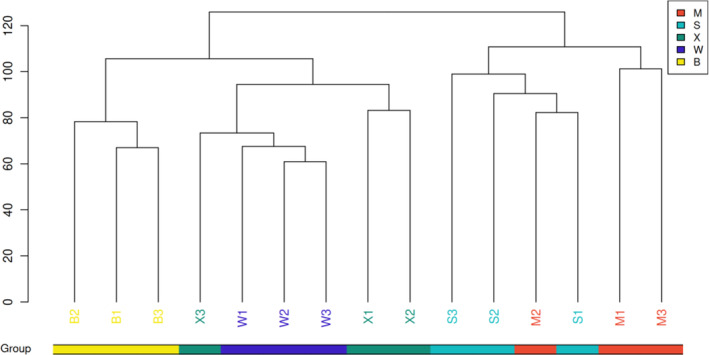
Population sample level clustering tree. In the legend of this figure, five different colors are used to represent the five cultivars, M for ‘Mahan’, S for ‘Shaoxing’, X for ‘Shoshoni’, W for ‘Wichita’, and B for ‘Pawnee’, the samples under the same cluster tree have higher similarity. In the later grouped figures, the classification of cultivars shown is the same as in this legend.

The distance matrix of 15 kernel samples from five *C. illinoinensis* cultivars was constructed, and sample similarity was evaluated using hierarchical clustering. The five *C. illinoinensis* were divided into two groups (Figure 2) with a certain correlation between the ‘Shaoxing’ and ‘Mahan’ ripe kernel samples. There was some similarity in the lipid cluster among ‘Pawnee’, ‘Shoshoni’, and ‘Wichita’ kernels, and the correlation between ‘Shoshoni’ and ‘Wichita’ was strong. Lipid structural composition in the nuts of ‘Shoshoni’ and ‘Wichita’ cultivars was highly similar, and the top 10 lipid types accounted for the largest proportion in the nuts (cf. Figure 1).

### 3.3 Multivariate statistical analysis

Because of dimension diversity and strong correlation among some variables, the traditional single variable analysis would not quickly, fully, and accurately mine the potential information inside the lipid group. Therefore, in the process of analyzing lipid metabolism data, it is necessary to collect and refine data multidimensionally by multivariate statistical analysis. Standard data were obtained by weight transformation of the experimental data, and multivariate statistical analysis was performed using principal component analysis and orthogonal partial least squares.

#### 3.3.1 Principal component analysis

New comprehensive characteristic variables generated by the linear combination of various weighted lipid variables represent the principal components, and these data were classified using principal component analysis. PCA is the linear superposition and accumulation of original variables. Since there was no intervention of external human factors, the obtained comprehensive variable model can fully reflect the original state of lipid metabolomics data, which is beneficial to grasp the data from an overall perspective. The principal component analysis of kernel samples of different *C. illinoinensis* cultivars in the same period should assist in clarifying cultivar differences.

**FIGURE 3:**
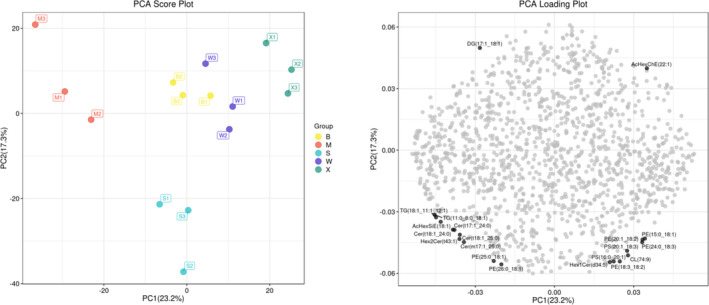
PCA analyses (a) Kernel sample clustering and dispersion of different cultivars, and (b) PCA loading diagram.

Model interpretability was 0.54 cum (>0.50 cum), indicating a strong degree of fitting and statistical data results that are true and effective. Sample point distributions show the clustering of the five cultivars with distinct separation of each cluster (Figure 3a). Among them, the ‘Pawnee’, ‘Wichita’, and ‘Shoshoni’ clusters are relatively close, indicating that they are compositionally relatively similar and the differences between them are relatively small. The four lipid molecules (PE, Cer, PS, and TG) make a strong explanatory contribution to the explanation of the original variables, contributing 6, 4, 2, and 2 metabolic molecules, respectively (Figure 3b). These four lipids dominate lipid composition of *C. illinoinensis* cultivar mature nuts and can be used as the main variables for evaluating *C. illinoinensis* oil quality.

#### 3.3.2 Orthogonal partial least squares

**FIGURE 4:**
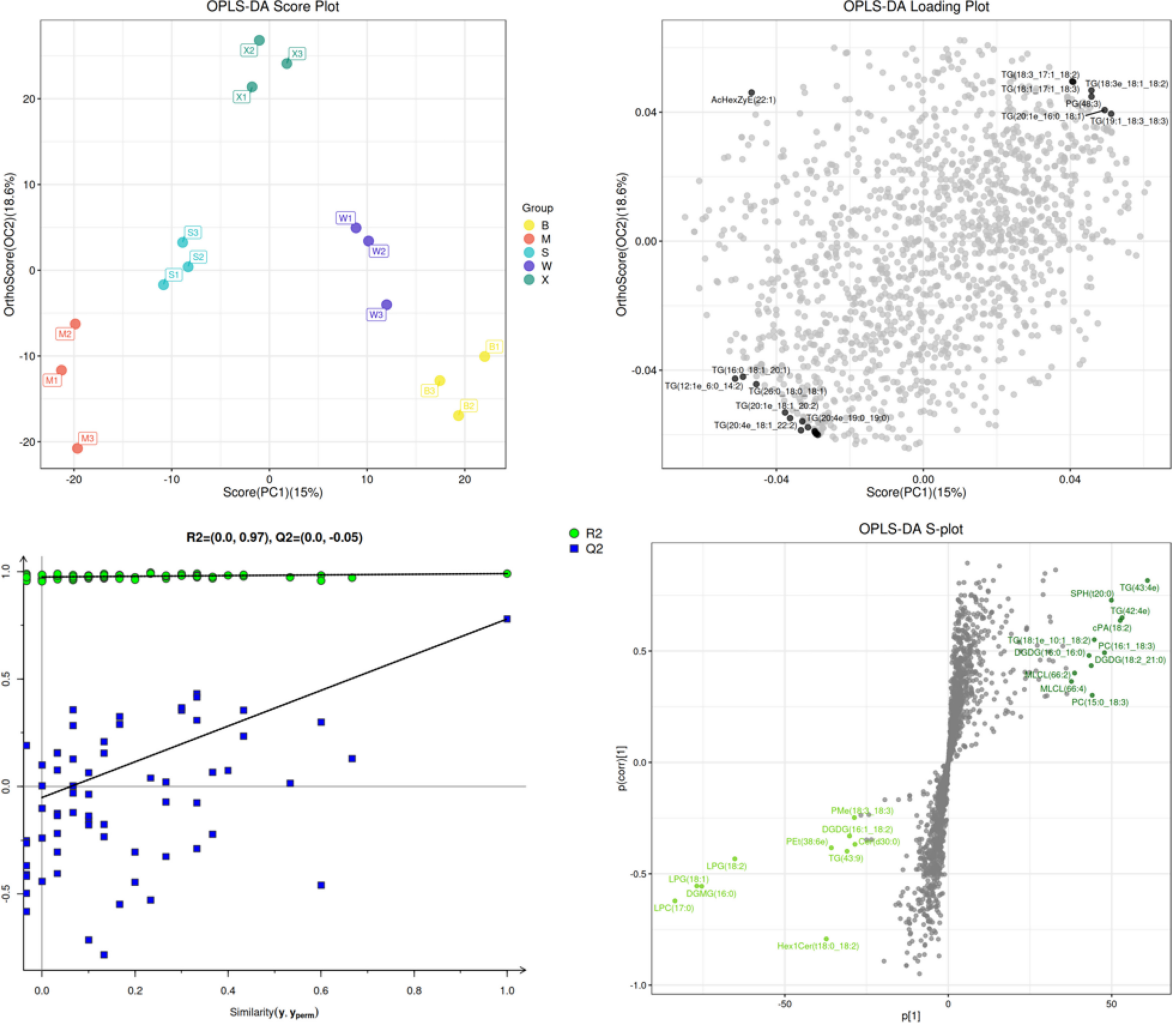
OPLS analyses (a) Kernel sample clustering and dispersion of different cultivars, (b) OPLS‐DA loading diagram, (c) OPLS‐DA permutation test, and (d) OPLS‐DA S‐plot analysis.

In lipidomics, OPLS is a data analysis method based on PLS, which decomposes the model matrix information into two categories using orthogonal signal correction technology and comprehensively evaluates the model classification effect according to individual component scores. As an extension of partial least squares, orthogonal partial least squares can clarify group differences. We used OPLS to establish a model of five *C. illinoinensis* kernel samples. The model predictive degree was 0.779 cum, and its explanatory power for X and Y data sets was 0.451 cum and 0.990 cum, respectively. Samples among the mature kernel groups of the five *C. illinoinensis* cultivars were relatively dispersed and the samples within each group clustered, indicating significant differences among different cultivars and high repeatability of kernel samples within the same group (Figure 4a). In the permutation test figure, the intersection point between the rightmost original blue point Q2 and the ordinate was <0, and all other Q2 points were lower than the original point, indicating that the model prediction was true and effective (Figure 4c). In the loading plot, TG accounted for up to 90% of the top 20 lipid molecules, indicating that TG is the dominant lipid in mature kernels of the five *C. illinoinensis* cultivars (Figure 4b). S‐plot analysis showed that TG, SPH, LPC, LPG, and DGMG were the most important lipids in the mature kernels of the five *C. illinoinensis* cultivars (Figure 4d).

Overall, PE and TG were the two most important lipid components in the mature nuts of the five *C. illinoinensis* cultivars, and PE was one of the phospholipids. Phospholipids will emerge with oil during oil extraction. Soybean oil has high phospholipid content, and its phospholipid is often used as an important source of plant phospholipid. In our study, the proportion of PE in *C. illinoinensis* mature kernels was relatively high, indicating that the kernels had potential in the extraction and development of vegetable oil. From a human health viewpoint, TG and PE content often reflect health status of the human body. High PE content is known to cause a fatty liver, and TG is often regarded as an indicator of abnormal blood lipid. Moreover, people with hyperlipidemia often have higher TG content. The relatively high proportion of lipids such as TG and LPC in mature *C. illinoinensis* nuts suggests that they may have unique potential in food nutrition and health care. Daily supplements and intake of *C. illinoinensis* nuts may improve blood circulation and prevent cardiovascular diseases in humans.

### 3.4 Analysis of differential lipids

#### 3.4.1 Differential lipid class correlation heat map

**FIGURE 5:**
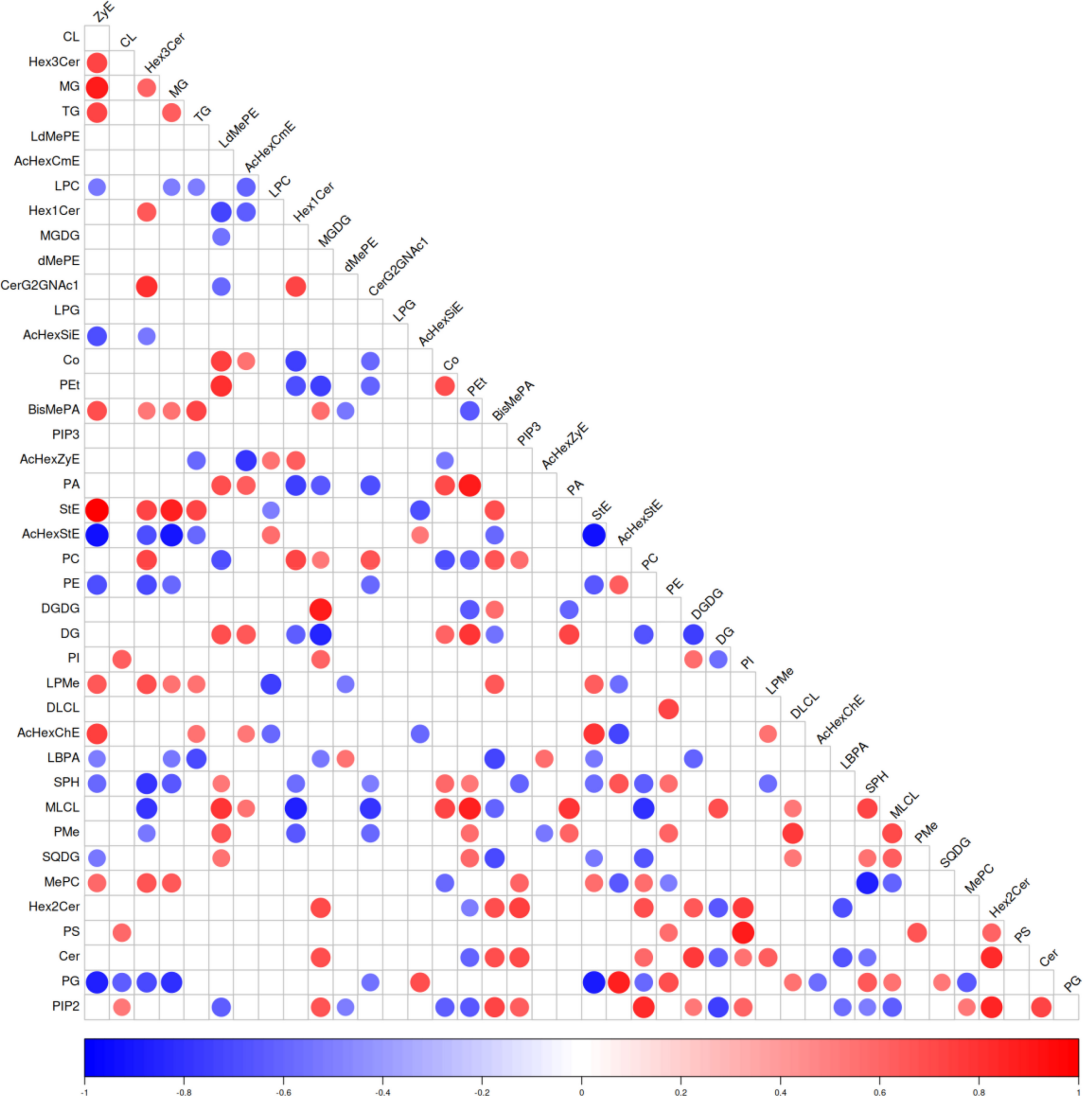
Differential lipid Class association heat map. In this figure, red indicates a positive correlation and blue indicates a negative correlation. The darker the color, the stronger the correlation.

Differences in 80 related lipids in mature nuts of the five *C. illinoinensis* cultivars were analyzed, and a class correlation heat map of different lipids was constructed (Figure 5). Pearson correlation coefficients were used to determine relationships between two lipids. Correlation coefficients vary from −1.0 and 1.0, the closer the value was to 1, the stronger the positive correlation between two lipids, and the closer to ‐ 1, the greater the negative correlation. Values approaching zero indicate a weak correlation between the two lipids and a low degree of association. There were 820 correlations between every two lipids, among which 127 were positive and 111 were negative (Figure 5). Further analysis showed that there were strong correlations between the same lipid subclasses, and there was a strong positive correlation between DGDG and MGDG in glycerolipids. The correlation coefficient between DG and MGDG or DGDG was close to −1, showing a strong negative correlation. The correlation coefficients between PS and PI, PA and PEt in phospholipids were red, and close to 1, showing a high degree of positive correlation. The correlation between AcHexStE and StE of sterol lipids was blue, and close to −1, indicating a high degree of negative correlation. Some lipids, such as DLCL, PIP3, and LPG, had very weak correlations with other lipids. Only PE was positively correlated with DLCL, indicating that correlations between DLCL and other lipids were weak or absent.

Relationships between different lipids were very complicated and lipid subclasses of the five cultivars showed only certain correlations. Changes in the major lipid classes lead to changes in other subclasses, and different subclasses show certain correlations. When the proportion and quantity of a certain type or a certain lipid change, the content of other lipids related to it will change accordingly, thus affecting the proportion of various lipids in the whole lipid group.

#### 3.4.2 Differential lipid class bar chart

**FIGURE 6:**
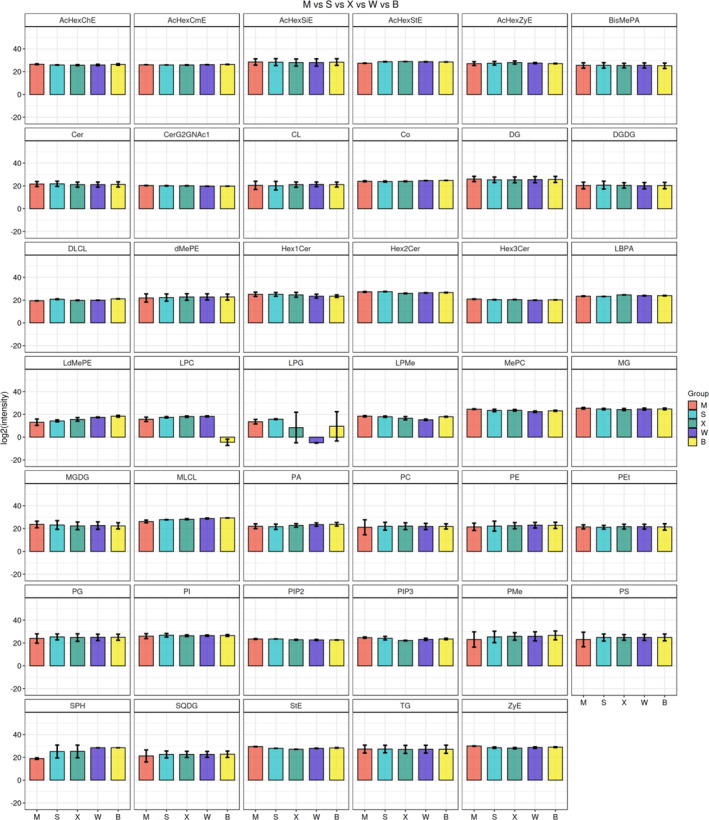
Histogram of differential lipid class classification. M vs. S vs. X vs. W vs. B in the top part of this figure represents the comparison of different lipids among the five cultivars of ‘Mahan’, ‘Shaoxing’, ‘Shoshoni’, ‘Wichita’, and ‘Pawnee’. Rectangle height in each histogram represents lipid signal strength, significant differences are reflected by histogram height difference between groups, and the error bar represents the standard error.

We selected the first 41 lipid types with significant differences in mature nuts of the five *C. illinoinensis* cultivars, and compiled histograms of Class classification of differential lipids (Figure 6). From the figure, lipid types and the number of substances in each lipid subclass varied among species, and in different cultivars, lipid types showed significant differences.

In ‘Mahan’, glycerolipid and sterol signal strength in mature kernels was higher than in the other four cultivars, which was reflected in MG, MGDG, StE, and ZyE lipid contents. In ‘Pawnee’ and ‘Shoshoni’ ripe nuts, phospholipid content was relatively rich, while ‘Pawnee’ had mainly high PA and PMe content. ‘Shoshoni’ had mainly high LBPA content (lysophospholipids), which have a role in regulation of human fat metabolism, can prevent the accumulation of intravascular cholesterol, and reduce blood viscosity. Sphingolipids and glycerophospholipids were relatively rich in ‘Wichita’ and ‘Shaoxing’. CL, LPC, and SPH signals in ‘Wichita’ were significantly higher than in the other four cultivars. PG, PE, PI, and LPG in glycerophospholipids were significantly higher in ‘Shaoxing’, and the LPG signal was significantly different from the other four cultivars. Overall, differences in the LdMePE signal among the five *C. illinoinensis* cultivars were obvious (Figure 6), and there was a significant height difference among cultivars. We suggest that cultivars can be preliminarily identified by analyzing their nut LdMePE content.

The LPC signal in ‘Pawnee’ was significantly lower than in the other four cultivars, and in ‘Wichita’ was significantly different from the other cultivars. The relatively low LPC contents in kernels of the two cultivars indicate that they may have a weak role in regulating serum cholesterol, and further confirm the conclusion that lipid subgroups under the lipid category are not positively correlated. Among the ceramide lipids, Hex1Cer and Hex3Cer signals in ‘Wichita’, Hex1Cer in ‘Pawnee’, and Hex2Cer in ‘Shoshoni’ were significantly lower than in the other cultivars. For ceramide lipids, this may indicate differences in degree of hydrolysis and amount of hydrolysis products in mature nuts of the 5 *C. illinoinensis* cultivars, as polymerization degree of each lipid subclass in the mature nuts of different cultivars were different.

Among the first 41 significantly different lipid types, 17 were found to have relatively uniform data distribution among and within groups, mainly concentrated in ceramides AcHex and HexCer and phosphatidylinositols PIP2 and PIP3. Ceramide was found to have obvious anti‐aging and skin moisturizing effects. As a water‐soluble lipid, its structure is similar to the substance that constitutes the human stratum corneum. After dissolving in water, it forms a network structure that prevents water dissipation, thus achieving a moisturizing effect. Additionally, increasing ceramide content in the human body helps to improve the cuticle, enhancing skin elasticity, and delaying aging. PI can promote calcium channel opening and play an important role in regulating cell signal transduction. Among individual lipid types, a relatively large dispersion of intragroup data was found for LPG in ‘Shoshoni’ and ‘Pawnee’, SPH in ‘Shaoxing’ and ‘Shoshoni’, and PMe in ‘Mahan’.

According to lipid difference analysis and quantitative analysis results, glycerolipid contents in mature nuts of the five cultivars were the highest, and other kinds of lipids and their corresponding subclasses were significantly different among different cultivars.

### 3.5 Lipid functional enrichment assay

**FIGURE 7:**
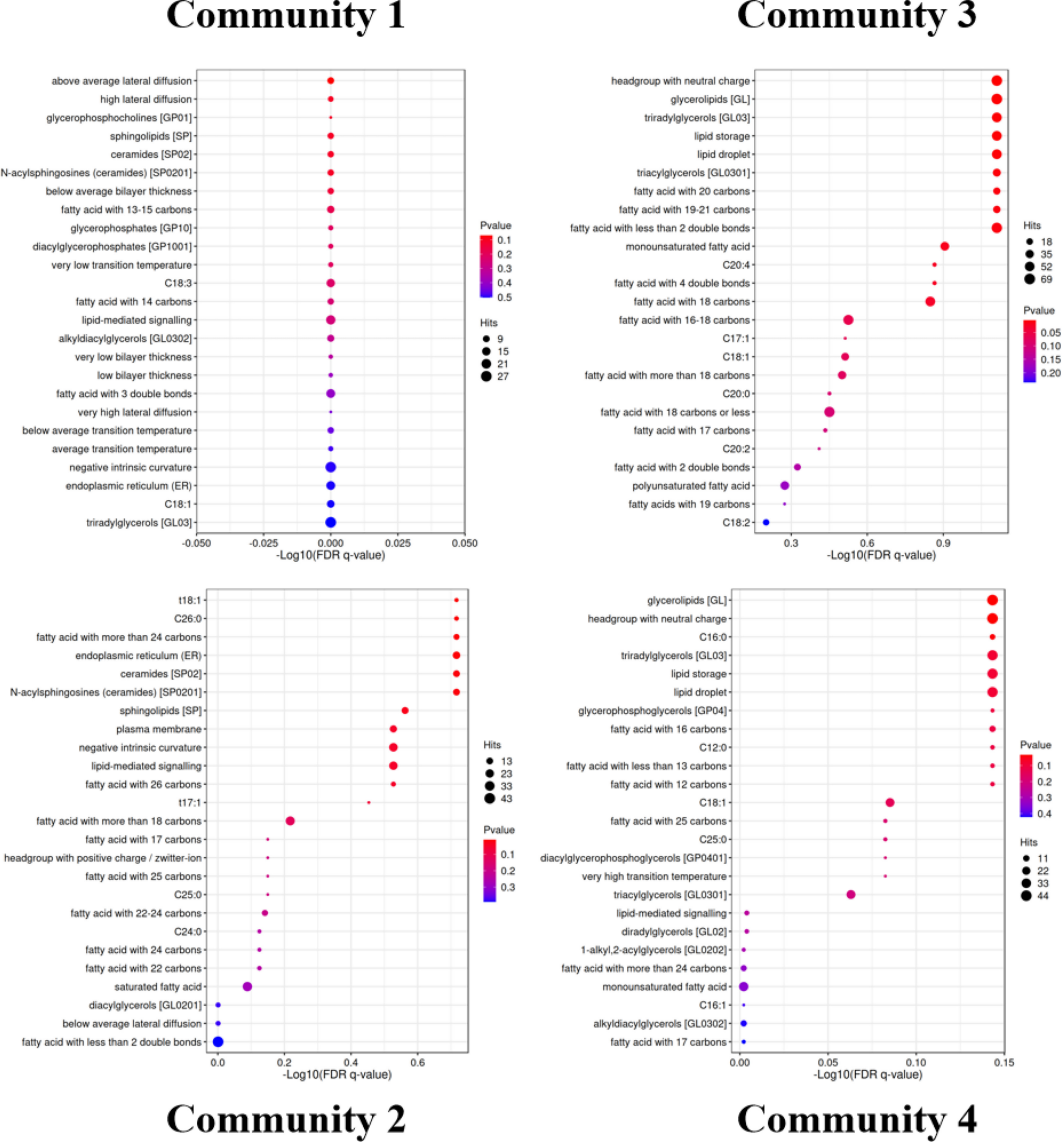
Lipid community. Enrichment analysis results are presented as LION bubble plots with the abscissa representing the −Log 10 value of the enriched FDR *q*‐value. The ordinate represents name, structure, and function of lipids in the enriched LION database. The color indicates the significance of the enrichment *p* value, the redder the color, the more significant the enrichment result. Bubble dot size indicates the number of differential lipids enriched into that entry, with larger dots indicating more enriched lipids.

The LION lipid ontology database was used for performing lipid functional enrichment analysis on different community classifications of all the differential lipids obtained under Leiden community classification calculation in the network analysis. Differential lipid functional enrichment analysis of mature nuts of the five *C. illinoinensis* cultivars was carried out to obtain four functional communities (Figure 7).

For the four functional communities, about 45 of the first 100 lipids show highly significant differences. In the mature kernels of the five *C. illinoinensis* cultivars, the number of different glycerolipid lipids was relatively the greatest. Glycerolipid is one of the main components of blood fat, which plays a very important role in blood transport and blood lipid storage. According to the previous quantitative analysis, TG content in the mature *C. illinoinensis* kernel was the highest, and sterol in phospholipid and lipid also existed in a limited amount. Blood lipid content can represent the degree of risk of atherosclerosis in humans. Intriguingly, the unique lipid type and number in ripened *C. illinoinensis* nuts suggest that they could be used as food for the prevention of cardiovascular and cerebrovascular diseases.

Additionally, some fatty acids, such as palmitic (C16: 0), oleic (C18: 1), linoleic (C18: 2), linolenic (C18: 3), arachidic (C20: 0), 11,14‐eicosadienoic (C20: 2), eicosatetraenoic (C20: 4), and tetracosanoic acid (C24: 0). This indicates that there are certain types and amounts of saturated and unsaturated fatty acids in mature *C. illinoinensis* kernels. Further scrutiny of Figure 7 showed that the oleic, linoleic, and linolenic acid bubble points were the largest, indicating that the number of differential lipids enriched in these three fatty acids was greatest in the kernel, and their content in *C. illinoinensis* was the highest. In community 3, the oleic acid bubble is red, and the enrichment result is significant, but in the first community, the oleic acid bubbles are blue, and the enrichment result is insignificant (Figure 7). The color difference of the fatty acids represented by the bubbles in the four communities reflect significant differences among the enrichment results, so we infer that certain correlations among some fatty acids in *C. illinoinensis* mature nuts, and the types and contents of fatty acids may be continuously converted during the nut ripening process.

## 4 CONCLUSIONS

Lipidomic analyses of five *Carya illinoinensis* cultivars identified 58 lipid classes in the mature kernel of each cultivar, and there was no significant difference in lipid composition among cultivars. Lipids of the five cultivars were mainly glycerolipids and phospholipids, and the glycerolipids accounted for about 65%. TG and DG were the main lipid types. Phospholipids accounted for >30%, mainly including Cer, PC, PE, PI, PS, PG, and PA. TG accounted for >50% of the mature kernels of all cultivars, and DG, Cer, PC, and PE ranked second to fifth in lipid content. There was a specific certain correlation between the ripe kernel samples of ‘Shaoxing’ and ‘Mahan’. There was some similarity in the lipid cluster among ‘Pawnee’, ‘Shoshoni’, and ‘Wichita’ kernels, and the correlation between ‘Shoshoni’ and ‘Wichita’ was the strongest. Multivariate analysis showed that TG, PE, Cer, and PS were the most important factors in the mature kernel of the five cultivars, and they made a significant contribution to kernel lipid composition, so they could be used as the main indicators for evaluating *C. illinoinensis* oil quality. Additionally, a series of fatty acids were detected in mature kernels of all cultivars, and oleic, linoleic, and linolenic acids were the most abundant. When the proportion and quantity of one kind of lipid changed, the content of other related lipids changed accordingly. Correlations between lipids also affected their interactions.

Huang et al. (2021) dynamically analyzed lipid composition during *C. cathayensis* embryo development and found that glycerolipid and glycerophospholipid nut contents were the highest, at 59.94% and 38.66%, respectively. In our research, *C. illinoinensis* mature kernel lipids were mainly composed of about 65% glycerolipids and >30% phospholipids. *Carya cathayensis* and *C. illinoinensis* lipid composition analysis showed that there was little difference in lipid classes between them, and both contained abundant glycerolipids. This may be because they belong to the same plant family and genus. Intriguingly, by analyzing the composition of both lipid subclasses, we found that PE, TG, PA, PI, and PS had the highest relative content in *C. cathayensis*. TG, DG, Cer, PC, and PE were found in *C. illinoinensis* ripe nuts. Lipid subclasses of the two showed obvious differences in the number of substances, which may be due to geographical isolation caused by origin problems (He et al., 2019). In the beginning, *C. cathayensis* was mainly distributed in Zhejiang Province of China (Fu et al., 2022), while *C. illinoinensis* originated from the United States and the south of Mexico (Jiao et al., 2022). Although the two are closely related, the geographical distance between their original locations is immense, and this geographical isolation may have led to a certain degree of reproductive isolation. Combined with these geographical differences, the two walnuts of the same genus showed great nutritional quality differences. Although *C. cathayensis* and *C. illinoinensis* differ in lipid composition, their mature nuts have a high protein and unsaturated fatty acid content, and therefore be beneficial for people to eat, effectively preventing atherosclerosis, reducing cholesterol, and enhancing brain function (Alasalvar & Bolling, 2015).

A characteristic high content and large proportion of phospholipids and glycerolipids in *C. illinoinensis* mature kernels provide a theoretical basis for the processing and utilization of plant and edible oils. The characteristics of rich TG, PC, and other lipids in various mature nut cultivars give them unique potential in food nutrition and health care. Appropriate daily supplements and intake of *C. illinoinensis* nuts may improve blood circulation and prevent cerebrovascular diseases, providing a new perspective for medicine and food homology development.

## AUTHOR CONTRIBUTIONS

**Zhe Zhao:** Formal analysis (lead); investigation (lead); methodology (equal); project administration (equal); writing – original draft (lead); writing – review and editing (equal). **Fei Wang:** Formal analysis (supporting); software (supporting); supervision (supporting); visualization (supporting); writing – original draft (supporting); writing – review and editing (supporting). **Tian Hu:** Data curation (supporting); formal analysis (supporting); software (supporting); supervision (supporting); writing – original draft (supporting). **Chun‐hua Zhou:** Conceptualization (supporting); funding acquisition (lead); project administration (lead); supervision (lead); writing – review and editing (equal).

## FUNDING INFORMATION

This study was financially supported by Forestry Science and Technology Innovation and Promotion Project of Jiangsu Province (LYKJ[2020]14) in China.

## CONFLICT OF INTEREST STATEMENT

The authors declare that they have no known competing financial interests or personal relationships that could have appeared to influence the work reported in this paper.

## Supporting information


Figures S1–S7

## Data Availability

Data are available on request from the authors.
