# Supplementary material for: Lipidomic analyses of five Carya illinoinensis cultivars
Source: Food Sci Nutr. 2023 Jul 31;11(10):6336–48. doi: 10.1002/fsn3.3572 (PMC10563669; doi:10.1002/fsn3.3572)
Supplement: Supplementary file 1 — Figures S1–S7 [file FSN3-11-6336-s001.pdf]

## 1. Box Plot of Differential Lipid Class

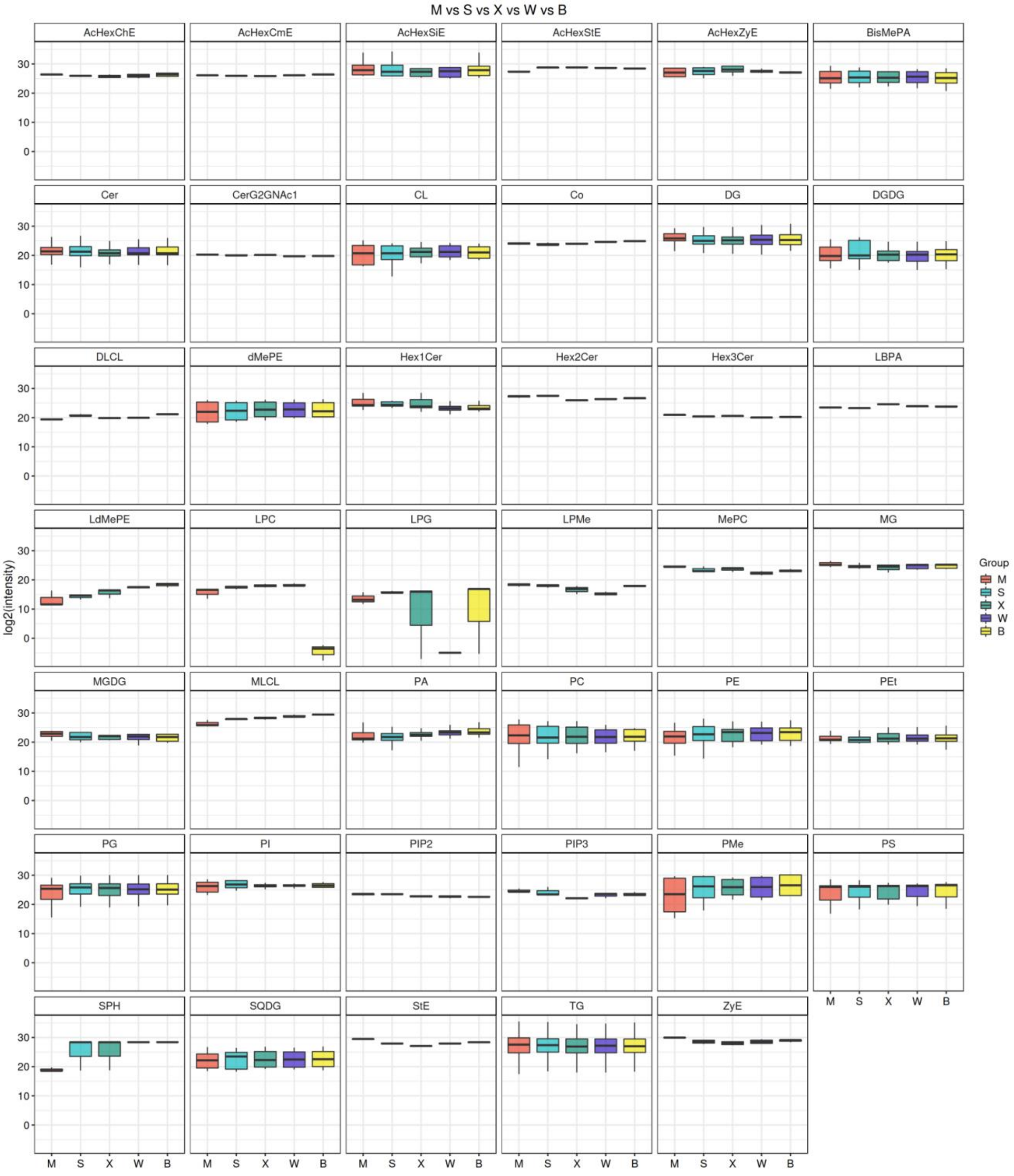


**Figure 1 Compartmentalized diagram of differential lipid Class**

## 2. Lipid structure characterization

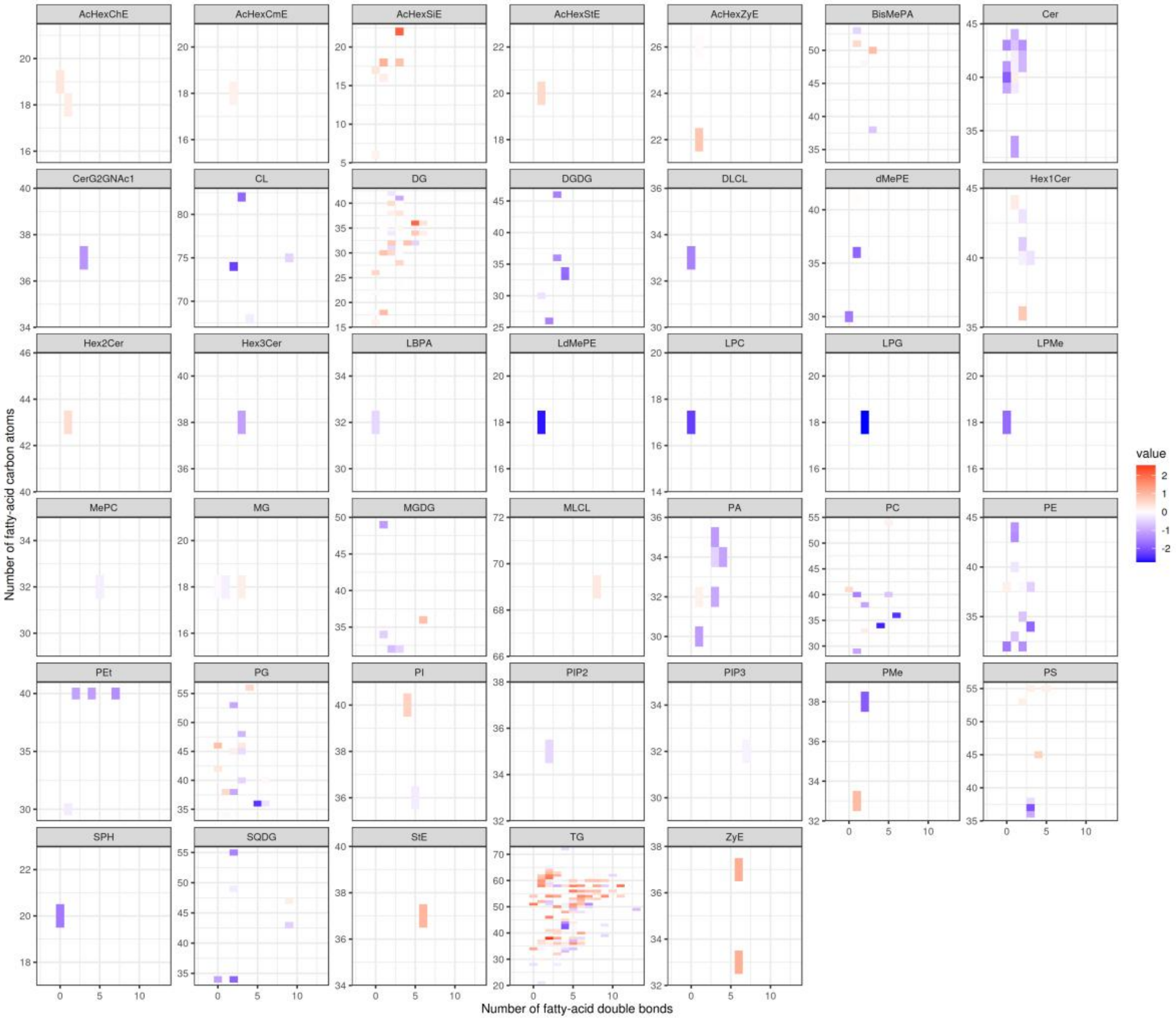


**Figure 2 Heat map of lipid structure characteristics of 'Pawnee'**

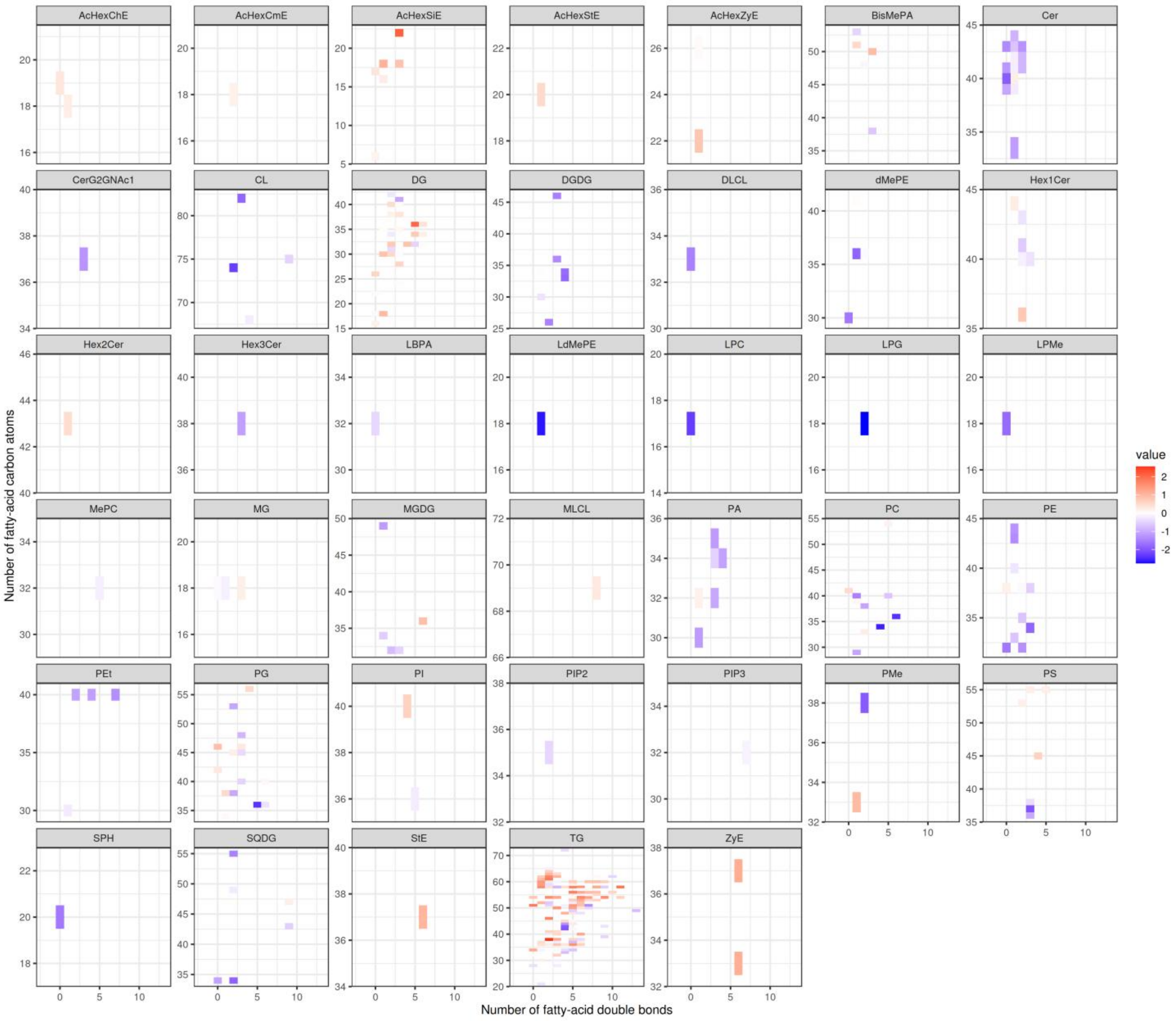


**Figure 3 Heat map of lipid structure characteristics of 'Mahan'**

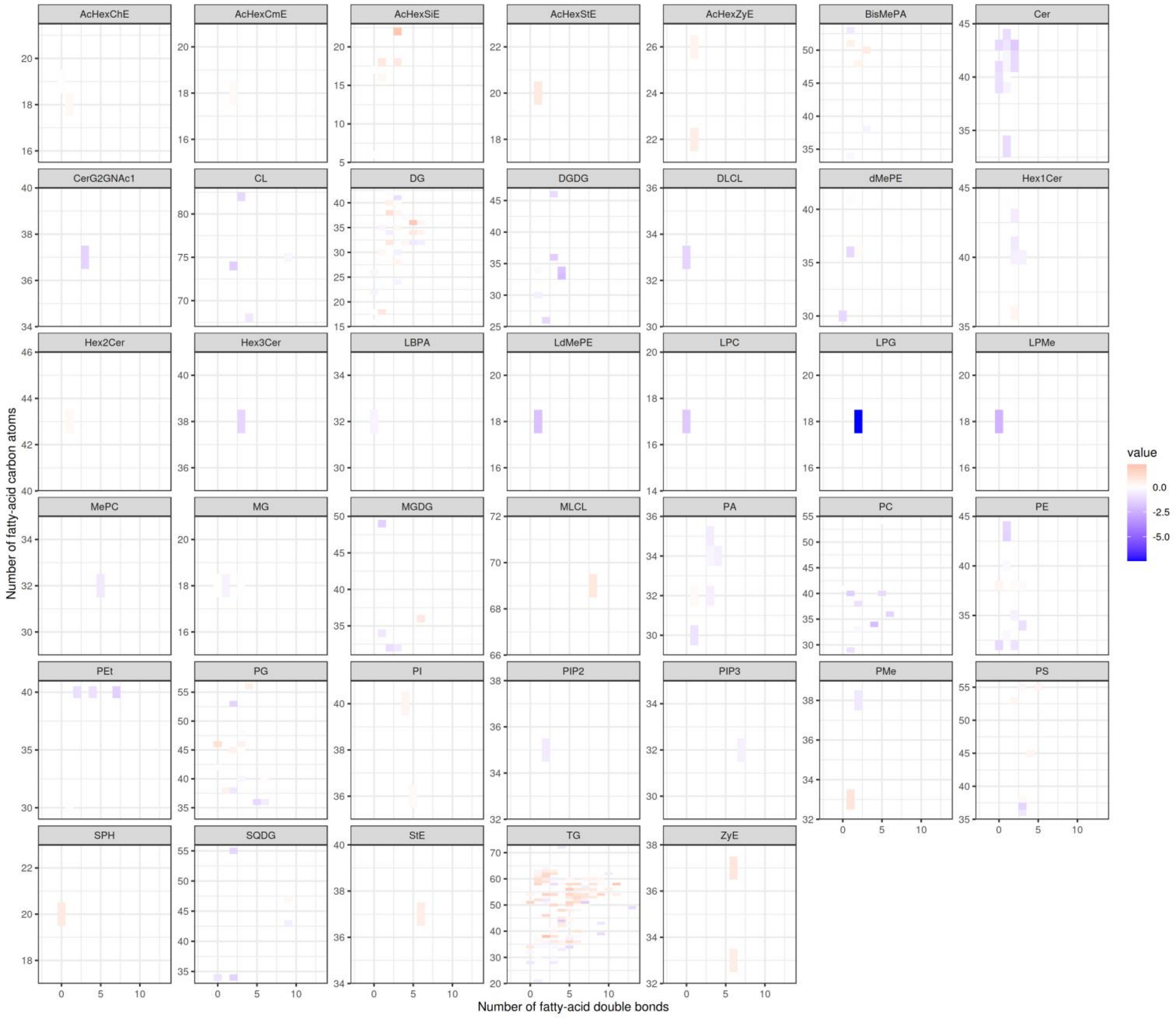


**Figure 4 Heat map of lipid structure characteristics of 'Wichita'**

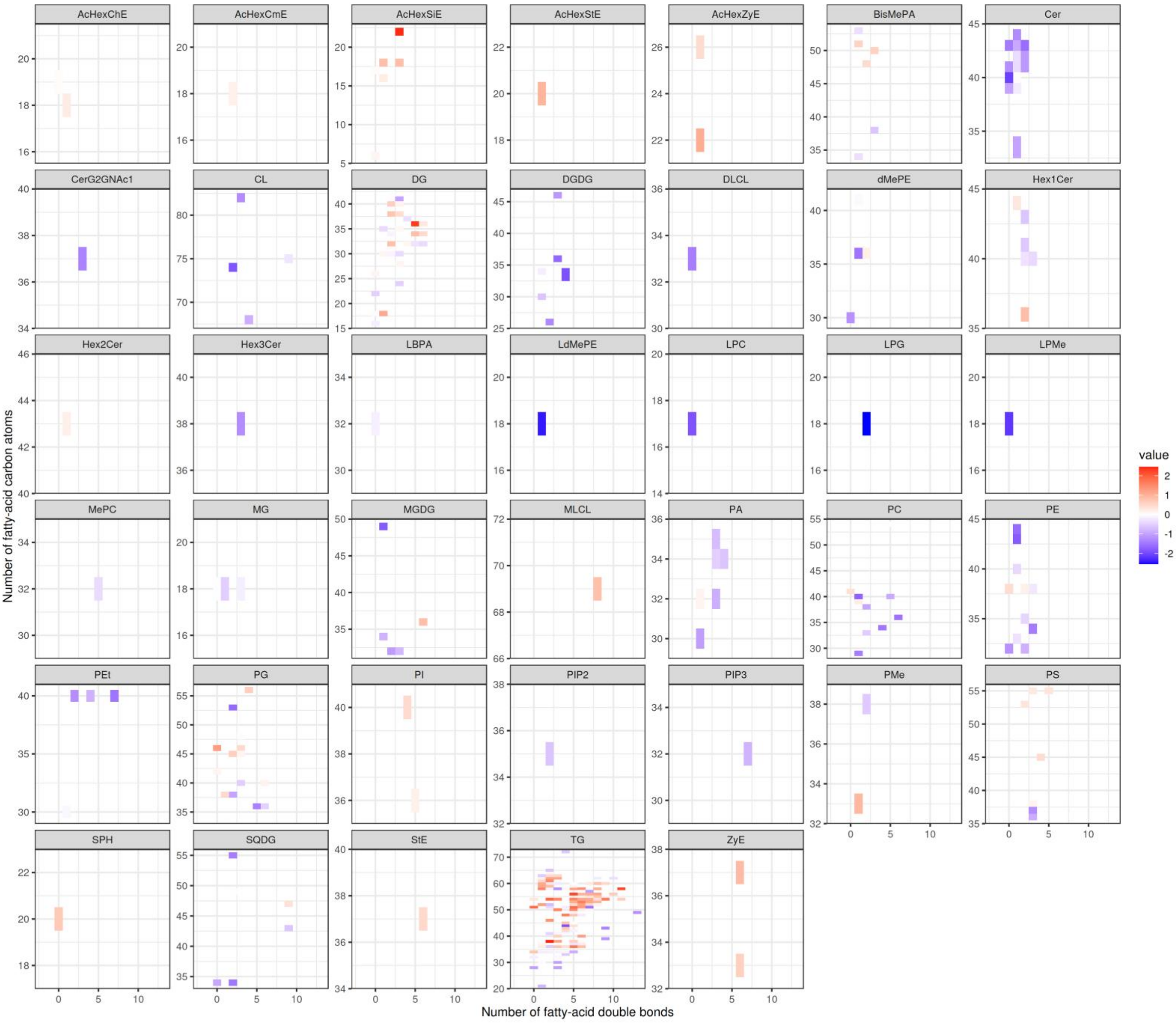


**Figure 5 Heat map of lipid structure characteristics of 'Shoshoni'**

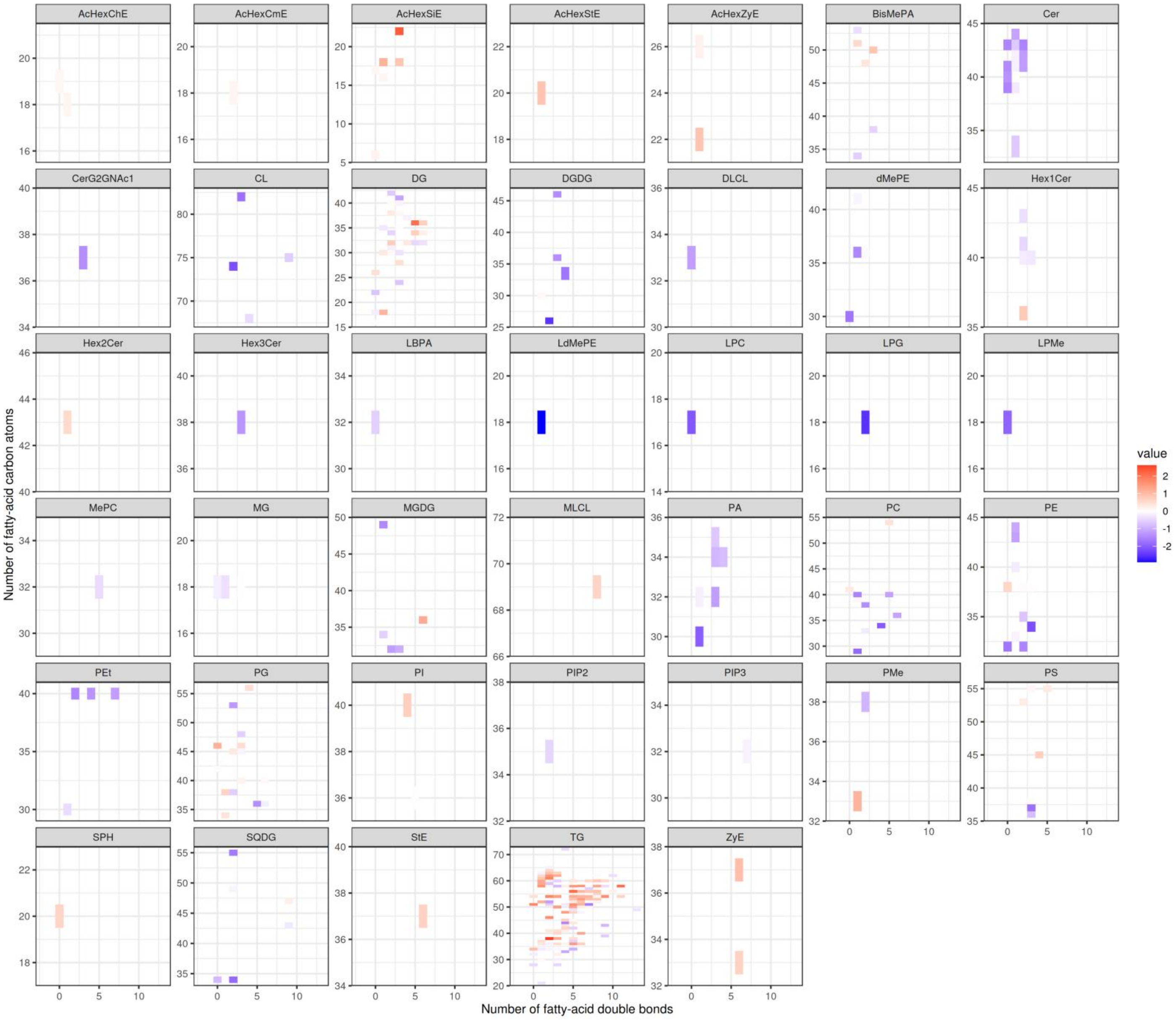


**Figure 6 Heat map of lipid structure characteristics of 'Shaoxing'**

## 3. Relevance Community Network Analysis

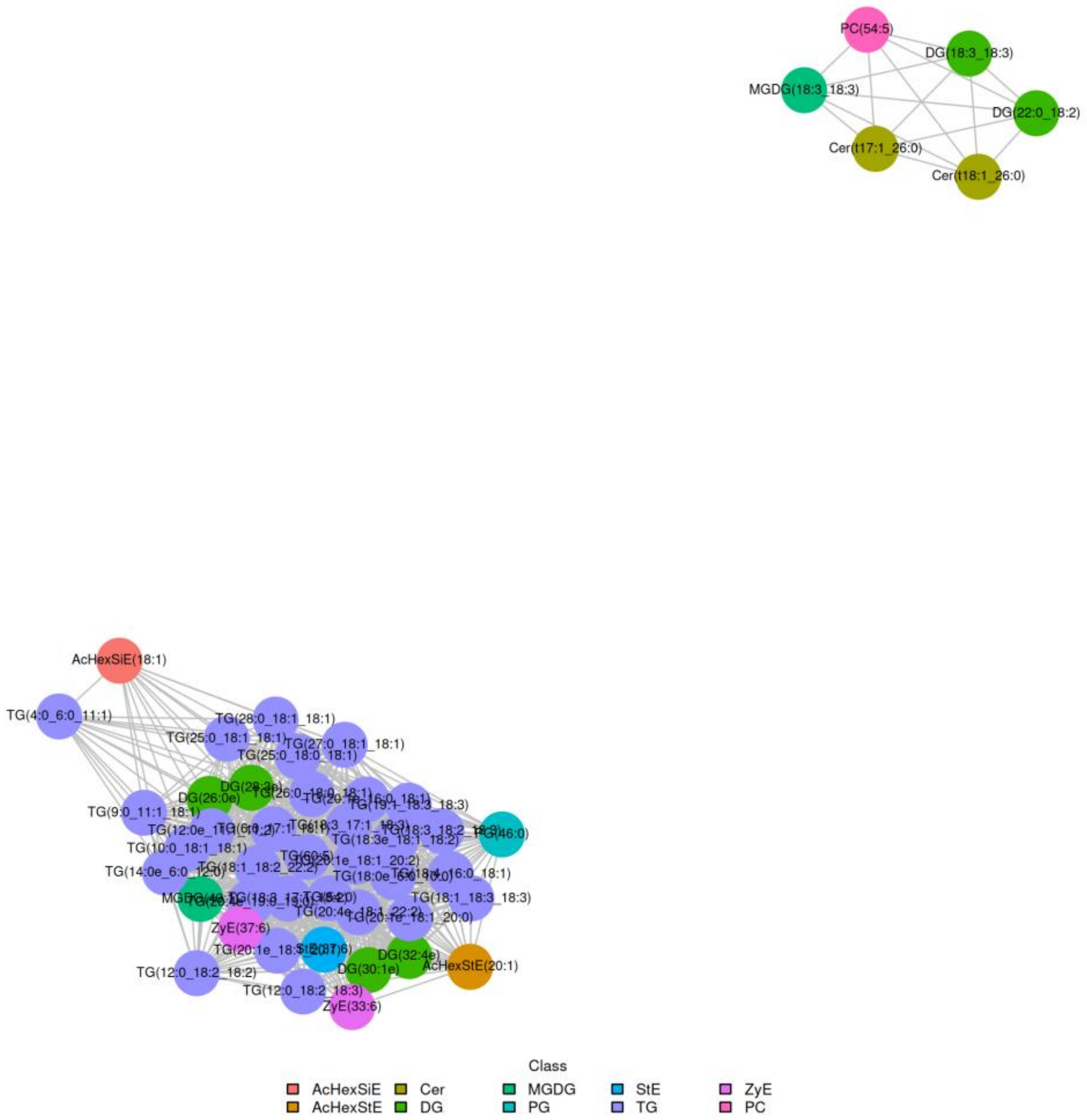


**Figure 7 Diagram of differential lipid correlation network**
